# Left Ventricle Biomechanics of Low-Flow, Low-Gradient Aortic Stenosis: A Patient-Specific Computational Model

**DOI:** 10.3389/fphys.2022.848011

**Published:** 2022-04-06

**Authors:** Andrew D. Wisneski, Yunjie Wang, Salvatore Cutugno, Salvatore Pasta, Ashley Stroh, Jiang Yao, Tom C. Nguyen, Vaikom S. Mahadevan, Julius M. Guccione

**Affiliations:** ^1^ Department of Surgery, University of California, San Francisco, San Francisco, CA, United States; ^2^ Thornton Tomassetti Life Sciences, Santa Clara, CA, United States; ^3^ Department of Engineering, Viale Dell Scienze, Universita degli Studi di Palermo, Palermo, Italy; ^4^ CATIA, Dassault Systèmes, Wichita, KS, United States; ^5^ Simulia, Dassault Systèmes Simulia, Johnston, RI, United States.; ^6^ Division of Cardiology, University of California, San Francisco, San Francisco, CA, United States

**Keywords:** aortic stenosis, finite elememt method, myofiber stress, ventricular function, aortic stenosis, realistic simulation, ventricle-aortic coupling

## Abstract

This study aimed to create an imaging-derived patient-specific computational model of low-flow, low-gradient (LFLG) aortic stenosis (AS) to obtain biomechanics data about the left ventricle. LFLG AS is now a commonly recognized sub-type of aortic stenosis. There remains much controversy over its management, and investigation into ventricular biomechanics may elucidate pathophysiology and better identify patients for valve replacement. ECG-gated cardiac computed tomography images from a patient with LFLG AS were obtained to provide patient-specific geometry for the computational model. Surfaces of the left atrium, left ventricle (LV), and outflow track were segmented. A previously validated multi-scale, multi-physics computational human heart model was adapted to the patient-specific geometry, yielding a model consisting of 91,000 solid elements. This model was coupled to a virtual circulatory system and calibrated to clinically measured parameters from echocardiography and cardiac catheterization data. The simulation replicated key physiologic parameters within 10% of their clinically measured values. Global LV systolic myocardial stress was 7.1 ± 1.8 kPa. Mean stress of the basal, middle, and apical segments were 7.7 ± 1.8 kPa, 9.1 ± 3.8 kPa, and 6.4 ± 0.4 kPa, respectively. This is the first patient-specific computational model of LFLG AS based on clinical imaging. Low myocardial stress correlated with low ejection fraction and eccentric LV remodeling. Further studies are needed to understand how alterations in LV biomechanics correlates with clinical outcomes of AS.

## Introduction

Aortic stenosis (AS) is the most common acquired heart valve disease in the developed world (Lindman et al., 2013). With the advent of transcatheter aortic valve replacement (TAVR), there has been increased attention to better understanding AS pathophysiology and how to optimally select patients for aortic valve replacement. Low-flow, low-gradient (LFLG) AS, first described by Hachicha et al., is a disease characterized by low aortic valve area but, given ventricular dysfunction, an elevated trans-valvular pressure gradient is lacking (Hachicha et al., 2007). It is estimated that up to 25% of all severe AS cases may be classified as LFLG (Hachicha et al., 2007; Pibarot and Dumesnil 2012; Clavel et al., 2016). This has raised challenges in the clinical management and diagnosis of patients suspected of having LFLG AS, with many diagnostic algorithms recommending dobutamine stress echocardiography to determine presence of LV contractile reserve (Pibarot and Dumesnil 2012; Pibarot and Clavel 2015; Clavel et al., 2016). Established evidence-based guidelines to determine diagnostic criteria for severe AS in presence of LFLG, and whom to select for aortic valve replacement, remains a topic of ongoing study (Nishimura et al., 2014).

The goal of aortic valve replacement, through TAVR or surgery, is to halt progression of and reverse pathologic left ventricle (LV) remodeling from chronic increased afterload of the stenotic aortic valve. We believe that a contemporary understanding of AS pathophysiology should encompass detailed analysis of ventricular function. Advancements in computational techniques and imaging have enabled creation of high-fidelity models of the human heart and LV (Sack et al., 2016; Sack et al. 2018a; Sack et al. 2020). We previously modeled aortic stenosis with a comprehensive human heart model to determine myocardial stress values in a non-LFLG AS case (Wisneski et al., 2020), with a model that was based on idealized ventricular geometry without any ventricular dysfunction. Detailed cardiac clinical imaging, and enhanced image processing techniques afford new opportunities to create patient-specific models to understand LV biomechanics.

We describe the first patient-specific computational model of the LV in a patient with LFLG AS. This model is derived from clinical cardiac computed tomography (CT) imaging which was obtained for TAVR planning purposes. While this study reports an initial case, this method should be scalable to permit greater numbers of patient-specific models to be generated. Future studies should correlate biomechanics data to disease severity, progression, and treatment outcomes.

## Methods

### Clinical Case and Image Processing

A 68-year-old man with co-morbidities of hypertension, hyperlipidemia, diabetes mellitus, psoriatic arthritis, chronic kidney disease, and coronary artery disease with prior percutaneous coronary intervention developed progressive AS, limiting his functional status. From transthoracic echocardiography, the LV ejection fraction of 25% was measured and eccentric LV geometry was noted. The mean pressure gradient across the aortic valve was 15 mmHg with an estimated aortic valve area of 0.8 cm^2^. LFLG AS was confirmed with dobutamine stress echocardiography. Coronary angiogram confirmed that his prior coronary stents were patent. The Society of Thoracic Surgeon’s mortality score was 11% for isolated surgical aortic valve replacement, rendering this patient a high-risk operative candidate, thus he was considered for TAVR.

**FIGURE 1:**
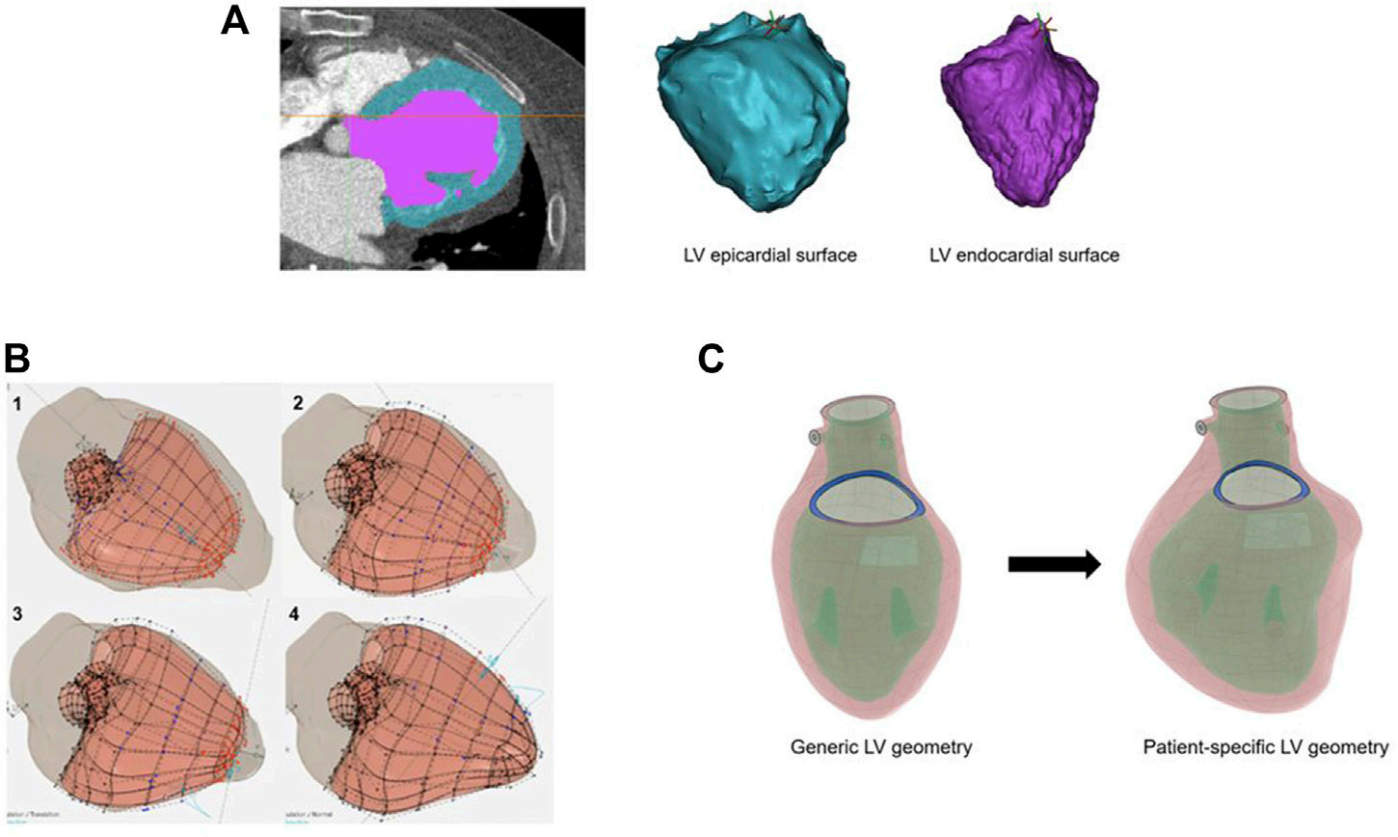
**(A)** Image segmentation of the left ventricle from computed tomography imaging. The left ventricle (LV) wall is in blue, and the LV cavity is in purple. At right are the three-dimensional surfaces representing the LV epicardial and endocardial surface boundaries. **(B)** The CATIA™ software allows rapid alignment of the generic model (salmon-colored model) to patient-specific imaging surfaces (left ventricle shadow overlay shown in gray). Alignment of the generic epicardial surface to patient-specific epicardial surface: 1) initial overlay, 2) alignment of the basal segments, 3) mid-wall segments, 4) apical segments. **(C)** Overview of the generic left ventricle geometry transformed to patient-specific geometry after application of the Smart Geometry processing.

Images from the diastolic phase of the ECG-gated CT scan were used to provide geometry for the computational model (Figure 1A). CT scan slices were 0.625 mm contiguous axial slices acquired on a GE Lightspeed VCT scanner (GE Healthcare, Chicago, Illinois, United States). All CT images were anonymized prior to analysis for research purposes, done in accordance with the institutional review board.

Segmentation of the LV, left atrium, and the aortic root was performed with Mimics version 2.1 (Materialise, Leuven, Belgium). Separate segmentation of the innermost surface of the LV chamber at the endocardium, and the outermost LV surface at the epicardium provided the LV wall geometry (Figure 1A).

### Computational Model

Our computational model platform, which has been described previously, provides realistic anatomy of the chambers and valves and accounts for multiple domains of cardiac function, including electrical activation, valve function and structure, myocardial material properties, myocardial microstructure and fiber orientation, and for blood flow (Carrick et al., 2012; Baillargeon et al., 2014; Sack et al., 2018a; Wisneski et al., 2021). The LV myocardium material model has been described in the aforementioned references. The ventricular model passive response uses the Holzapfel and Ogden anisotropic hyperelastic model (Holzapfel and Ogden 2009). The deviatoric response is governed by the following strain energy potential:

$$\Psi_{dev}=\frac{a}{2b}\exp\left[b\left(I_{1}-3\right)\right]+\sum_{i=f,s}\frac{a_{i}}{2b_{i}}\left\{\exp\left[b_{i}\left({\left(I_{4i}-1\right)}^{2}\right)\right]-1\right\}+\frac{a_{fs}}{2b_{fs}}\left[\exp(b_{fs}I_{8fs}^{2}-1\right]$$ (1)

Eight material parameters a *,* b *,* $a_{f}$ *,* $b_{f}$ , $a_{s}$ *,* $b_{s}$ *,* $a_{fs}$ *,* $b_{fs}$ , and four strain invariants $I_{1}$ *,* $I_{4f}$ *,* $I_{4s}$ *,* and $I_{8fs}$ define Eq. 1. For these simulations, a=3.354 kPa, b=7.08 , $a_{f}=2.501$ kPa, while the remaining parameters were set to null. The strain invariants are derived from the isochoric right Cauchy-Green tensor:

$$\bar{C}=\;{\bar{F}}^{T}\bar{F}=J^{-2/3}C=\;J^{-2/3}F^{T}F$$ (2)

*F* is the deformation gradient, J is the determinant of the deformation gradient, J=det(F)  and $\bar{F}$ is the isochoric part of the deformation gradient where $\bar{F}=\;J^{-1/3}F$ and $\det\left(\bar{F}\right)=1$ . The strain invariants can now be defined as:

$$I_{1}=tr\left(\bar{C}\right),\;I_{4f}=\;f_{0}\cdot \left(\bar{C}f_{0}\right),\;I_{4s}=\;s_{0}\cdot \left(\bar{C}s_{0}\right),\;I_{8fs}=f_{0}\cdot \left(\bar{C}s_{0}\right)$$ (3)

Terms *f*_*0*_ and *s*_*0*_ are orthogonal vectors in the fiber and sheet direction in the reference configuration. The volumetric response is governed by:

$$\Psi_{vol}=\;\frac{1}{D}\;\left(\frac{\left(J^{2}-\;1\right)}{2}-\ln\left(J\right)\right)$$ (4)

Where J is the third deformation gradient invariant, and *D* is the multiple of the bulk modulus $\left(D=\;\frac{2}{K}\right).$

The active myocardial tissue response is represented as a time-varying elastance model (Guccione and McCulloch 1993; Guccione et al., 1993; Holzapfel and Ogden 2009; Carrick et al., 2012; Wenk et al., 2012; Genet et al., 2016; Sack et al., 2018b; Wisneski et al., 2020):

$$\sigma_{af}\left(t,E_{ff}\right)=\;\frac{T_{\max}}{2}\;\frac{Ca_{0}^{2}}{Ca_{0}^{2}+\;ECa_{50}^{2}\;\left(E_{ff}\right)}\left(1-\cos\left(\omega \left(t,E_{ff}\right)\right)\right)$$ (5)

With functions defined as:

$$ECa_{50}\left(E_{ff}\right)=\;\frac{Ca_{0\max}}{\sqrt{e^{B\left(l\left(E_{ff}\right)\right)-l_{0}}-1}}\;$$ (6)

$$\omega \left(t,E_{ff}\right)=\;\pi \frac{t}{t_{0}}\;when\;0\leq t\;<\;t_{0}$$ (7a)

$$\omega \left(t,E_{ff}\right)=\;\pi \frac{t-\;t_{0}+\;t_{r}\left(l\left(E_{ff}\right)\right)}{t_{r}}\;when\;t_{0}\leq t\;\leq \;t_{0}+\;t_{r}\left(l\left(E_{ff}\right)\right)$$ (7b)

$$\omega \left(t,E_{ff}\right)=0\;when\;t>t_{0}+\;t_{r}\left(l\left(E_{ff}\right)\right)$$ (7c)

$$t_{r}\left(l\right)=ml+b$$ (7d)

$$l\left(E_{ff}\right)=\;l_{r}\sqrt{2E_{ff}+1}\;$$ (7e)

*T*_*max*_ is the maximum allowable active tension and is multiplied by terms regulating calcium concentration and the time course of the contraction. These two terms are dependent on the sarcomere length *l*. Parameters were set as follows: $T_{\max}=135.7$ kPa, $Ca_{0}=4.35umol/l$ , $Ca_{0\max}=4.35µmol/l$ , $m=1.0489sµm^{-1}$ , b=−1.429s , $B=4.750µm^{-1}$ , $l_{0}=1.58µm$ . $l_{r}$ is the sarcomere length in the unloaded state, and was assumed to vary linearly from 1.78 μm at the endocardium to 1.91 μm at the epicardium.

**FIGURE 2:**
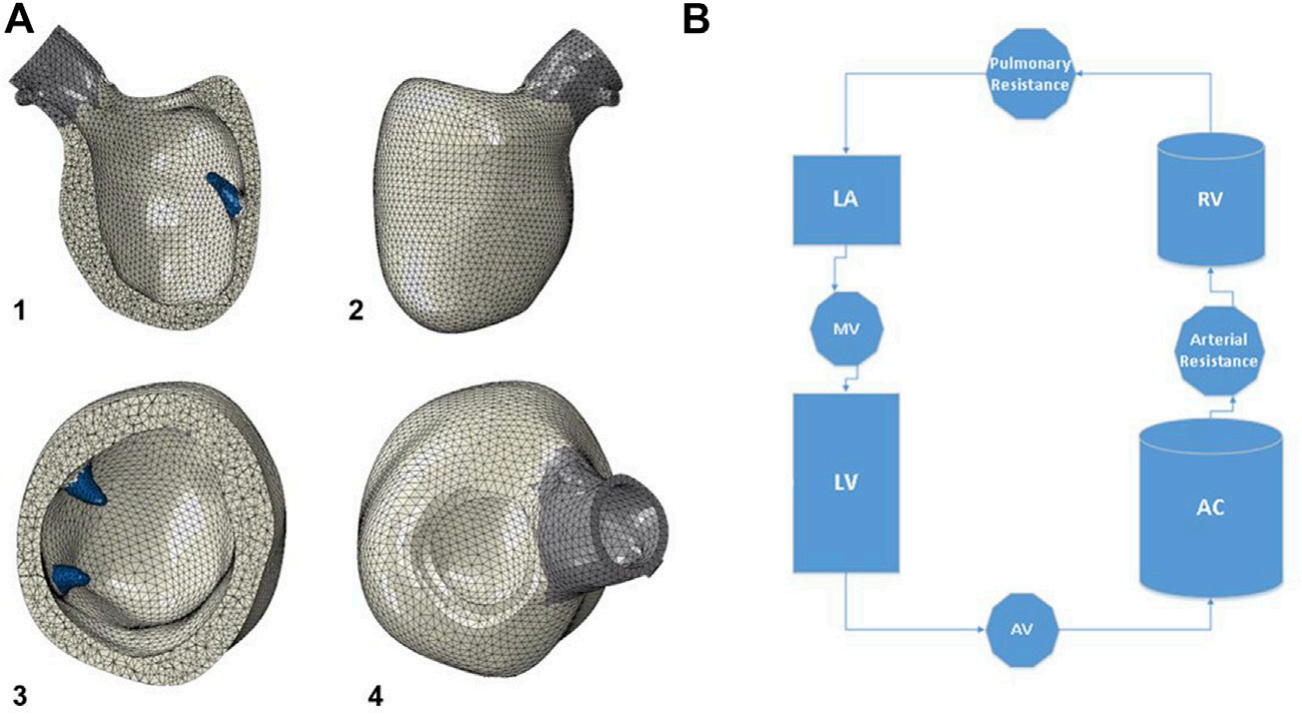
**(A)** The model geometry is then meshed, with the left ventricle consisting of 91,000 ten-noded tetrahedral elements. The papillary muscles are shown in blue, and were excluded from biomechanical analysis of the left ventricle. 1) anterior cutaway, 2) short axis cutaway, 3) posterior view, 4) superior view. **(B)** Diagram of the circulatory model connected to the left ventricle for cardiac cycle simulations. Valves are assigned resistance values, and chambers are assigned elastances. LV: left ventricle, AV: aortic valve, AC: arterial chamber, RV: right ventricle, LA: left atrium, MV: mitral valve.

The idealized geometry of the heart model was adapted to the patient-specific geometry with the aid of CATIA™ software (3D Systems, Johnston RI, United States) (Figure 1B). Groups of nodes representing the LV wall could be moved in sync to line up with surface geometry from image segmentation. This enabled efficient transformation from generic LV geometry to that of patient-specific geometry (Figure 1C). With the focus of this study being LV biomechanics, the LV was represented by a mesh of 91,000 individual solid elements, each consisting of a 10-noded tetrahedron bound by the surfaces obtained from the CT imaging segmentation (Figure 2A). Although portions of the left atrium and LV outflow tract were included in the model geometry, their primary purpose was to serve as boundary conditions for the LV model. The left atrium, LV outflow tract, and papillary muscles were excluded from biomechanical analysis. The LV was subdivided into 17 distinct segments in the basilar, mid-wall, and apical regions guided by the American Heart Association topographic classification system (Cerqueira et al., 2002).

The model was connected to a lumped-parameter virtual circulatory system for cardiac-cycle simulations run in Abaqus® FEA (Simulia, Johnston, RI, United States) (Figure 2B). AS was created by increasing the aortic valve resistance to generate a trans-valvular pressure gradient and elevated LV chamber pressures over the cardiac cycle (Wisneski et al., 2020). An iterative process was used to tune the model to the patient’s circulatory system and LV physiology based on echocardiographic and catheterization data. Cardiac cycle simulations were run with automated adjustments to myocardial material properties and systemic vascular resistances/compliances, which permitted simulation results to optimally replicate patient-specific physiology. An acceptable steady state was achieved until further cycles produced <5% variation in chamber pressures compared to the prior cycle. For the final model, the following system parameters were used: aortic valve resistance (AV) 5e− 9 MPa*s/mm^3^, arterial resistance 1.35e+ 02 MPa*sec/mm^3^, pulmonary vascular resistance 8e+ 0 MPa*sec/mm^3^, mitral valve resistance 2e+ 0 MPa*sec/mm^3^, arterial compliance (AC) 1.0e+ 07 mm^3^/MPa, pulmonary compliance (PC) 7.99e+ 06 mm^3^/MPa.

The results of LV stress (kPa) and strain along the direction of the myofibers were obtained at end-diastole and peak systole, and reported as mean ± standard deviation. The t-test was used for statistical comparison of continuous variables.

## Results

**FIGURE 3:**
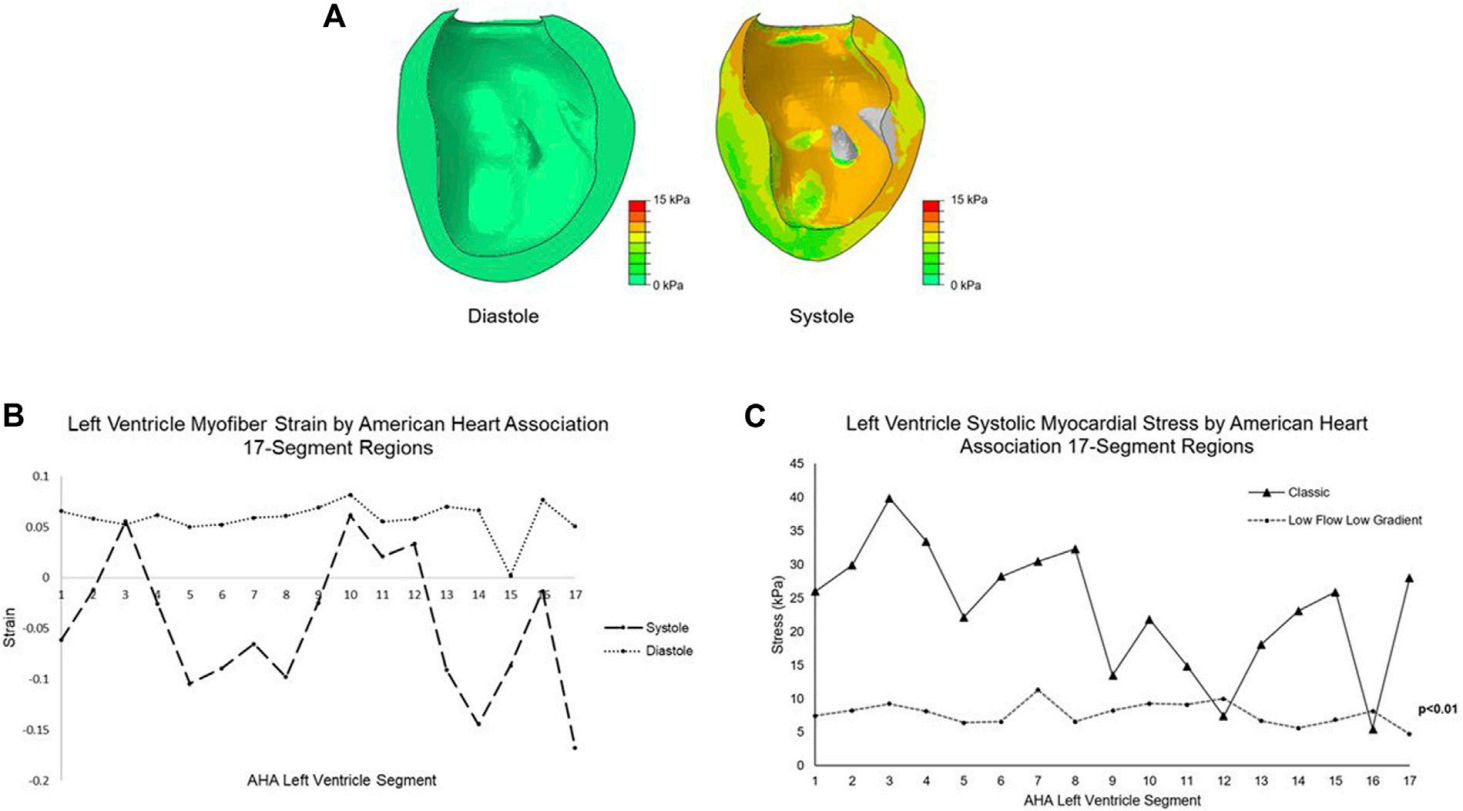
**(A)** Color plots of the left ventricle myocardial stress (kPa) at end diastole and peak systole. The papillary muscles are excluded from biomechanical analysis. **(B)** Mean myofiber strain at diastole and systole for the LFLG model by American Heart Association 17-segment left ventricle classification system. **(C)** The mean systolic stress of each left ventricle segment by American Heart Association classification system for the LFLG model compared to the idealized left ventricle model with classic severe AS. Lower magnitude stress values and low variation among the segments are found in the LFLG model. Segments 1-6 represent the basal aspect, segments 7–12 the LV mid-wall, and 13–17 the apical region. LFLG: low-flow, low-gradient; LV, left ventricle; AS, aortic stenosis.

Global LV peak systolic myocardial stress and strain were 7.1 ± 1.8 kPa and −0.07 ± 0.12; global LV end diastolic stress and strain were 0.24 ± 0.17 kPa and +0.07 ± 0.04 (Figures 3A–C). Further division by American Heart Association segment classification yielded mean basal region systolic stress of 7.7 ± 1.8 kPa, mean mid-wall region systolic stress of 9.1 ± 3.8 kPa, and mean apical region systolic stress of 6.4 ± 0.4 kPa. Myocardial systolic stress for each individual segment is plotted in Figure 3C. The patient-specific simulation achieved close correspondence to clinically measured parameters, listed in Table 1.

**TABLE 1:** Comparison of patient clinical parameters compared to simulation results.

Physiologic parameter	Patient measured	Simulation result
LV ejection fraction	25%	23%
LV systolic pressure	128 mmHg	118 mmHg
LV diastolic pressure	12 mmHg	6 mmHg
Aortic systolic pressure	116 mmHg	109 mmHg
Aortic diastolic pressure	45 mmHg	50 mmHg
Mean pressure gradient across aortic valve	15 mmHg	17 mmHg
Peak pressure gradient across aortic valve	25 mmHg	23 mmHg

Abbreviations: LV, left ventricle.

## Discussion

This study describes the first patient-specific computational model of the LV in a patient with LFLG AS, and the biomechanics results of myocardial stress and strain. This model was based on imaging obtained for TAVR planning purposes, and the use of specialized software enabled a generic LV model to be rapidly adapted to patient-specific geometry and physiology. The ability to create accurate computational models of the LV in AS facilitates the study of “LV-aortic coupling”, whereby aortic valve pathology is linked to LV function for a more complete understanding of the disease (Ikonomidis et al., 2019).

This model represents an advancement in computational investigations in two respects: 1) a patient-specific model of the LV can now be readily obtained from clinical imaging, rather than dedicated research-specific imaging, and 2) specialized software permitted efficient creation of a highly detailed mesh. Previously, weeks of effort were required for a single user to create highly detailed patient-specific ventricular geometry, whereas the CATIA™ software enabled it to be done by one user in approximately one and a half days. With this LV model having a high degree of detail represented by 91,000 elements, validated computational material properties and physiology, there is great potential to use clinical cardiac imaging for future studies of LV biomechanics in AS.

Our group previously published the myocardial stress associated with “classic” severe AS, where a mean transvalvular pressure gradient ≥40 mmHg exists, using a model of idealized LV geometry with normal LV function (Wisneski et al., 2020). Systolic stress of the LV was 16 ± 10 kPa. In contrast, systolic myocardial stress in the LFLG model is substantially reduced with a narrower standard deviation. We attribute this finding to the reduced LV function of the LFLG model, coupled with eccentric hypertrophy from pathologic remodeling. The globe-shaped ventricle creates a more uniform stress distribution. Breakdown of the classic AS LV model into the American Heart Association 17 segments demonstrated a relatively wider variation in segment stress with a range of 5.5–39.9 kPa as shown in Figure 3C. In the LFLG mode, the range across the LV segments is much narrower at 4.7–11.3 kPa. Comparison of LV segment mean systolic stresses yielded a significant difference with *p* < 0.01. The LFLG model’s eccentric hypertrophy with a dilated ventricle may explain the pronounced difference in stress distribution among the two models.

A computational modeling study by Lee et al. on LV geometry after surgical ventricular restoration for systolic heart failure concluded that a more spherical ventricle shape reduced myocardial stress magnitude and produced a more uniform stress distribution (Lee et al., 2013). The eccentric geometry of the LV in our LFLG model resulted in a similar finding when the stress magnitudes and distribution were compared to those of the normal, more ellipsoidal LV geometry in the classic AS model. It could be theorized that future ventricular biomechanics analysis showing reduced stress range throughout the LV may serve as biomechanical evidence of remodeling with reduced LV function. We acknowledge that in-depth analysis is limited by a sample comparison of one representative model from these two sub-types of AS, and that greater numbers of patient-specific models will be required for more definitive conclusions to be drawn.

There is a growing body of literature addressing the complexity of diagnosing severe AS when LFLG or suspected LFLG is encountered. Although a patient may be thought to have LFLG AS, many studies have used stroke volume index to help differentiate true low-flow, low-gradient AS from normal flow, low-gradient AS (Hachicha et al., 2007; Herrmann et al., 2011; Adda et al., 2012; Pibarot and Dumesnil 2012). This has highlighted limitations in use of LV ejection fraction as a sole indicator of true LV function. Research has demonstrated alternative indicators of LV dysfunction can be present in patients whose ejection fraction remains in the normal range. Adda et al. assessed ventricular longitudinal strain by speckle-tracking echocardiography in a cohort of patients with severe AS and normal ejection fractions. They concluded that compared to patients with normal flow, low-gradient AS, patients with LFLG AS had more severe stenosis with lower mean aortic valve areas, higher systemic afterloads, and decreased LV function with reduced basal longitudinal strain (Adda et al., 2012). Herrmann et al. found that patients with low-gradient severe AS had higher degrees of myocardial fibrosis and decreased longitudinal strain despite a preserved LV ejection fraction (Herrmann et al., 2011). Reliance on ejection fraction alone may miss subtle signs of LV dysfunction. Through further investigation with patient-specific models, we envision that biomechanics analysis will permit detection of the early signs of LV dysfunction.

A study by Shavik et al. applied computational simulation techniques to replicate the physiology of heart failure with preserved ejection fraction (HFpEF), whereby decreased longitudinal strain exists with a normal ejection fraction (Shavik et al., 2021). Through their framework, the variables of ventricular geometry, chamber size, blood pressure, and ventricular strains were altered based on sets of clinically measured patient-specific data. To adequately replicate HFpEF, the combination of depressed myocardial contractility coupled with increased afterload was required. While clinically distinct entities, LFLG AS and HFpEF may share some commonalities in the initial set of conditions that trigger the chronic pathologic remodeling.

Our results include strain along the direction of myofibers, a microscopic tissue-level organization of the myocardium. The differences between the LV segment strains at diastole and systole (Figure 3B) indicate the complex dynamics of ventricular contraction. This can be correlated to the known ventricular dysfunction this patient has, as several segments appear to contribute minimally to systolic LV contraction.

Myofiber orientation varies transmurally with a helix angle spanning −60° at the endocardium to +60° at the epicardium relative to the short axis of the heart (Walker et al., 2005; Carrick et al., 2012; Wenk et al., 2012; Genet et al., 2014; Genet et al., 2016; Sack et al., 2018b; Dabiri et al., 2018; Wisneski et al., 2020). Specialized imaging techniques such as diffusion-tensor and displacement encoding with stimulated echoes (DENSE) magnetic resonance imaging can be used to measure myofiber strain *in-vivo* (Bayer et al., 2012; Moulin et al., 2021). However, magnetic resonance imaging is more time consuming than CT imaging and is not routinely used for TAVR planning purposes. Computational models with myocardial material models accounting for myofiber orientation will be able to provide myofiber strain data throughout the LV. Myofiber strain should be differentiated from the strain reported in many clinical echocardiographic studies, such as global longitudinal strain, which describes deformation relative to the long axis of the heart. Reduced strain in LFLG AS, whether at the myofiber or global LV level, likely stems from the same mechanism of LV dysfunction.

The systolic stress and strain profile from the American Heart Association 17 segment classification can provide a unique biomechanical ‘footprint’ for a patient’s LV, provide a snapshot of LV performance, and help to categorize a patient’s disease severity in future investigations.

### Limitations

The study’s main limitation is that it only encompasses a single patient, limiting our ability to draw broader conclusions on LV biomechanics for LFLG AS. Future studies with greater numbers of AS patients will need to correlate biomechanics results with the severity of AS and LV dysfunction. Greater numbers of patient-specific models for LFLG AS versus “classic” elevated gradient AS will need to be compared as well. For computational efficiency, the model encompassed only the left heart, omitting the right atrium and ventricle. However, this case of isolated AS did not involve right heart disease; future cases that have biventricular dysfunction or diseases that affect the right heart should be modeled with both ventricles. Additionally, clinically measured data (ejection fraction, LV and aortic pressures) are used to calibrate the model, and close correlation was achieved. A patient’s physiology is expected to exhibit normal variation in daily life (i.e., heart rate, blood pressure) and thus we believe the model can yield useful data as long as the model results replicates key physiologic parameters within an acceptable range of the clinically measured values.

In our model, AS was created by increasing the aortic valve resistance parameter in the computational circulatory model, rather than creating a physical representation of calcium on the aortic valve restricting leaflet opening. Since our goal was to study the impact of AS on LV biomechanics, it was not necessary to create the physical representation of aortic stenosis, which permitted greater computational efficiency. This preliminary investigation demonstrates the feasibility of creating a patient-specific computational model from clinical cardiac imaging by modification of an existing LV model platform. There is great potential to generate biomechanics data to help elucidate the pathophysiology of LV dysfunction in AS.

## Conclusion

We describe the first patient-specific LV model in a case of LFLG aortic stenosis and the LV biomechanics results obtained. Compared to idealized LV geometry and normal ventricular function, reduced LV stress, an initial observation of globally reduced LV stress, was quantified. To translate patient-specific computational modeling to the clinical setting, future studies of larger AS populations should correlate biomechanics results with disease progression, ventricular dysfunction, and outcomes after aortic valve replacement. Data beyond traditional flow-derived metrics and ejection fraction should be incorporated into clinical assessment of AS and determination of who should receive aortic valve replacement. With the widespread adoption of TAVR, CT imaging obtained for pre-TAVR planning should yield abundant clinical imaging that can be used to create patient-specific models.

## Data Availability

The raw data supporting the conclusion of this article will be made available by the authors, without undue reservation.
